# Supplementary material for: Altered microRNA Transcriptome in Cultured Human Liver Cells upon Infection with Ebola Virus
Source: Int J Mol Sci. 2021 Apr 6;22(7):3792. doi: 10.3390/ijms22073792 (PMC8038836; doi:10.3390/ijms22073792)
Supplement: Supplementary file 1 [file ijms-22-03792-s001.zip › Supplementary_File/D_ KEGG_Analysis_Results/16-30nt_pathway_Makona-24h-Huh7_vs_Control-24h-Huh7_up.mature_mirna_targets/hsa_EnrichmentScoreDotPlot.pdf]

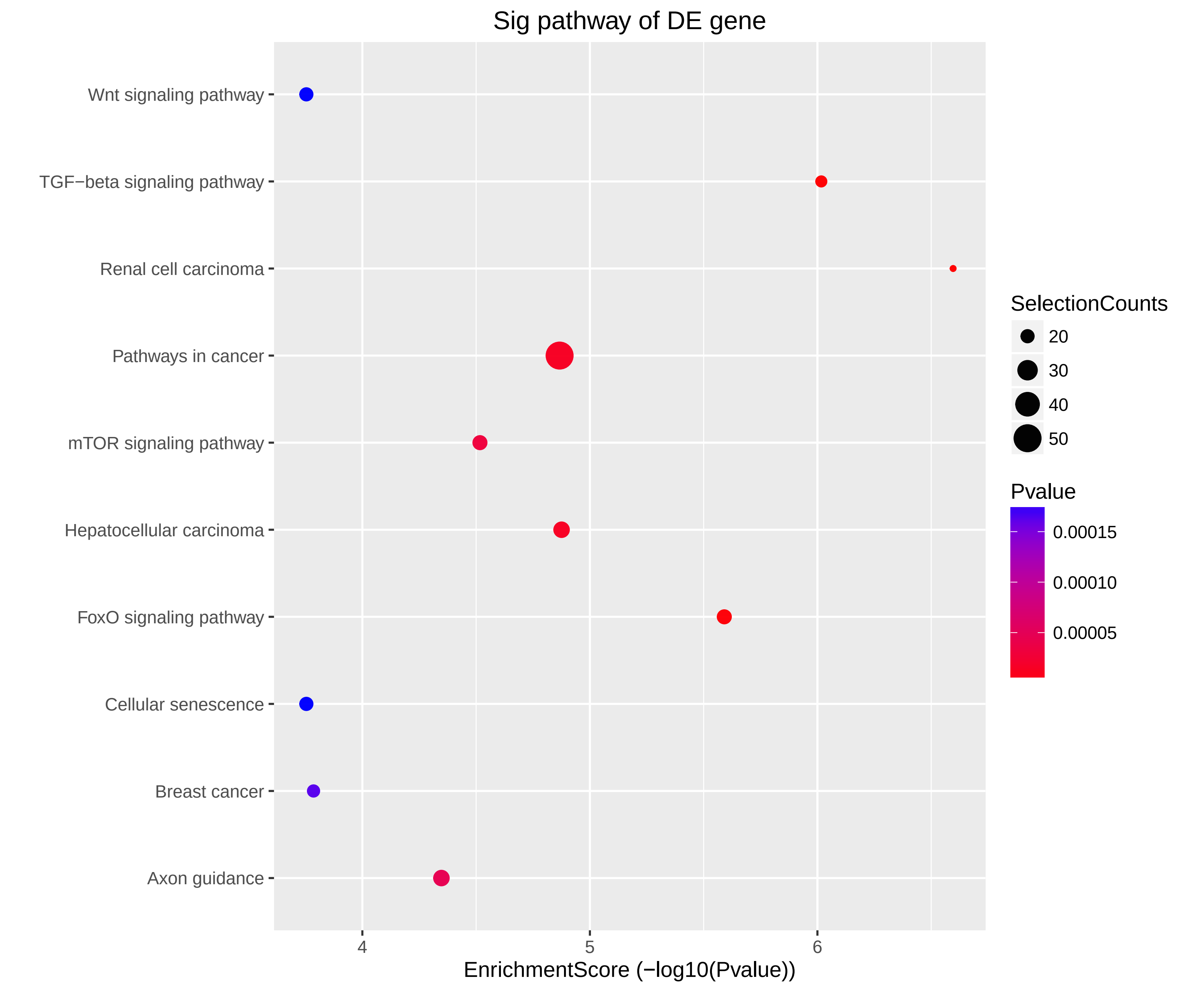
Sig pathway of DE gene
Wnt signaling pathway
TGF−beta signaling pathway
Renal cell carcinoma
Pathways in cancer
mTOR signaling pathway
Hepatocellular carcinoma
FoxO signaling pathway
Cellular senescence
Breast cancer
Axon guidance
4
5
6
EnrichmentScore (−log10(Pvalue))
SelectionCounts
20
30
40
50
Pvalue
0.00015
0.00010
0.00005
